# What It Takes to Manage Change: A Qualitative Study of Healthcare Managers’ Role Perceptions in Supporting and Sustaining TeamSTEPPS Implementation

**DOI:** 10.2147/JHL.S584769

**Published:** 2026-04-30

**Authors:** Robin Lüchinger, Katherine Blondon, Noelle Junod Perron, Marie-Claude Audétat

**Affiliations:** 1 Unit of Development and Research in Medical Education, University of Geneva, Geneva, Switzerland; 2 Medical Directorate, University Hospitals of Geneva, Geneva, Switzerland; 3 Interprofessional Simulation Center, University of Geneva, Geneva, Switzerland; 4 University Institute of Family and Child Medicine, University of Geneva, Geneva, Switzerland

**Keywords:** interprofessional collaboration, healthcare leadership, organizational change, implementation science, TeamSTEPPS, qualitative research

## Abstract

**Introduction:**

Sustaining interprofessional collaboration in healthcare settings requires more than formal change functions such as designated roles. It depends on leadership commitment, professional values, and structural alignment. While local champions play a key role in implementation, the role, perceptions and strategic involvement of institutional leaders remain underexplored.

**Methods:**

This qualitative study investigates how institutional leaders at the Geneva University Hospitals conceptualized and enacted their roles in the implementation of TeamSTEPPS framework. We conducted semi-structured interviews with 11 participants, including physician and nursing leaders and quality coordinators. Data were thematically analyzed using a predefined multi-level framework encompassing individual, inter-individual, and inter-group perspectives.

**Results:**

Our findings show that at the individual level, leaders’ role perceptions were strongly influenced by their professional backgrounds, values, and institutional positioning. Physician leaders leaned toward strategic oversight, while nursing leaders and quality coordinators emphasized practical application and staff commitment. Despite shared collaborative practices, participants faced constraints such as limited time, unclear role expectations, and institutional silos. At the inter-individual level, the local champions’ training enhanced interprofessional trust and skills. Success was associated with locally adapted strategies, participative leadership structures, and visible institutional support. At the inter-group level, tensions between promoted institutional values and operational reality such in communication and resource allocation hindered sustained engagement.

**Conclusion:**

Effective implementation of collaborative practices depends on leaders’ ability to align institutional goals with frontline realities. Supporting this alignment requires clearer role definitions, dedicated coordination, and strategic integration of change processes. A participative leadership approach is essential to foster ownership, coherence, and sustainability in healthcare transformation efforts.

## Introduction

In an era of continuous transformation, healthcare systems are under increasing pressure to adapt and innovate to ensure the delivery of high-quality care. Hospitals face the persistent challenge of implementing effective change initiatives while navigating clinical, organizational, and systemic constraints.^1^,^2^ To meet the growing demands of patients and stakeholders, healthcare institutions must engage in the ongoing improvement of care processes and professional practices across all specialties. This requires not only the assimilation of emerging evidence and technologies but also the continuous development of healthcare professionals’ competencies and collaborative skills.^3^

The successful implementation of change initiatives such as collaborative practices in healthcare settings depends on individual, team-based, and organizational factors.^4^,^5^ Effective change management requires a strategic approach that aligns institutional goals with local practices and values.^6^ In this context, individuals who operate at the intersection of strategic vision and frontline reality play a pivotal role in facilitating innovation.^7^,^8^ They are often referred to as “champions” and are recognized as important facilitators for the successful implementation of practices.^9^ These change champions are recognized not only for their commitment to improvement but also for their ability to mobilize teams, foster engagement, and navigate complex organizational dynamics.^10^ Their influence often stems from their formal institutional roles, professional legitimacy, and diverse career trajectories which collectively position them as credible agents of change.^11^ However, the sole presence of a champion does not ensure the success of a change initiative. Sustainable transformation relies on collaborative work practices that are co-constructed by multidisciplinary teams. Co-construction happens when team members, by discussing, arguing and negotiating the innovation to implement, become active implementation stakeholders in the process.^12^ These collaborative efforts are strengthened by shared values such as mutual respect, solidarity, and a commitment to patient-centered innovation. Participatory approaches to change foster collective ownership of projects. Embedded in an institutional culture, participatory approaches help transcend traditional professional silos.^13–15^ To be successful, such dynamics depend on a clear organizational vision, alignment of professional objectives, and the availability of appropriate resources.

The institution itself plays a central role in supporting implementation of collaborative practices by setting expectations, fostering alignment, and supporting the champions. Thus, institutional strategy is the prerogative of institutional leaders. While they are key enablers of coordination and motivation within their departments, project management often remains secondary to their primary responsibilities being first and foremost related to patient care. As a result, projects are often (unintentionally) deprioritized or delegated. Despite their strong motivation and commitment to improving collaborative practices, local champions lack support, guidance, and legitimization from the whole hierarchy to achieve lasting impact.^16^,^17^ In most situations, middle management (ie ward managers, fellows) occupy a critical position in this process, serving as translators of institutional strategy and facilitators of local implementation.^18^ However, still little is known about how their leaders with institutional decision-making power (ie at departmental or service levels) perceive and enact their role in supporting collaborative practice development.^19^ Are they consciously engaging with their role as facilitators of change, is their involvement more reactive and ad hoc? How do they understand their responsibility in bridging institutional ambitions with the realities of frontline teams? Exploring these empirical questions is essential to better understand the relational and strategic work of leadership in driving the implementation of change.

This study seeks to explore how institutional leaders in hospital settings conceptualize their role in the implementation of collaborative practices (TeamSTEPPS). By institutional leaders, we integrated heads of department (physician and nursing leaders) and quality and safety coordinators. Specifically, our study aims to investigate the extent to which those leaders are aware of and intentionally engage with their function as agents of alignment, support, and coordination in change processes. By examining their perspectives and actions through Doise’s conceptual framework, this research contributes to a deeper understanding of leadership consciousness in complex healthcare environments.

## Method

### Design

We conducted a qualitative research project within the University Hospitals of Geneva (HUG) between April and December 2024. This study was grounded in an interpretivist qualitative paradigm, aiming to explore how leaders construct meaning around their implementation roles.^20^ Qualitative design is especially suited for uncovering how individuals make sense of complex, value-laden roles in dynamic systems.^21^,^22^ This level of introspection is particularly valuable when studying healthcare leadership, which often involves implicit negotiations of legitimacy, identity, and responsibility.

### Setting

The Geneva University Hospitals (HUG) comprise a network of eleven hospitals and twenty care sites, offering a total of 2095 beds throughout the canton of Geneva, Switzerland. Serving a community of over 500,000 individuals, the HUG employs more than 13,000 staff members, positioning it as a key healthcare provider in French-speaking Switzerland. The institution includes ten medical divisions and one non-medical division. Its core missions encompass the provision of secondary and tertiary care (with 255,500 patients treated and 65,000 hospitalizations recorded in 2024), research (964 scientific publications in 2024), and education (1776 medical students enrolled and 176 federal diplomas in human medicine awarded in 2024).

To enhance interprofessional collaboration and improve teamwork within various care settings, the University Hospitals of Geneva have been implementing the TeamSTEPPS framework since 2021. This framework offers a set of evidence-based tools for fostering collaboration, along with guidelines for successful implementation.^23^,^24^ It comprises five dimensions, namely leadership, team structure, situation monitoring, mutual support, and communication. For example, for communication, TeamSTEPPS “SBAR” tool (Situation, Background, Assessment, Recommendations) may help structure handoffs. To implement TeamSTEPPS, the institution chose to integrate staff members in the process. This process comprised 3 phases: 1) identification and selection of champions with division or departmental leaders, 2) training of the champions and preparation for implementation (eg TeamSTEPPS 2-days Champion training, support from the institutional project group), and 3) implementation of certain tools by the champions and follow-up with debriefing of the project group. The newly formed championing teams could include quality and safety coordinators. These individuals are detached from the hierarchical line and have a cross-departmental role between decision-makers and the field. The approach selected by the institution was advantageous in that it created a collaborative team composed of healthcare professionals from different disciplines. Moreover, with the help of the project group, each team had the opportunity to reflect on the selected tools and adjust them to their local needs. Institutional leaders with the help of the TeamSTEPPS project group determined the specific implementation focus and timeline, leading to diverse experiences in how local champions carried out the project.

### Participants

**Figure 1:**
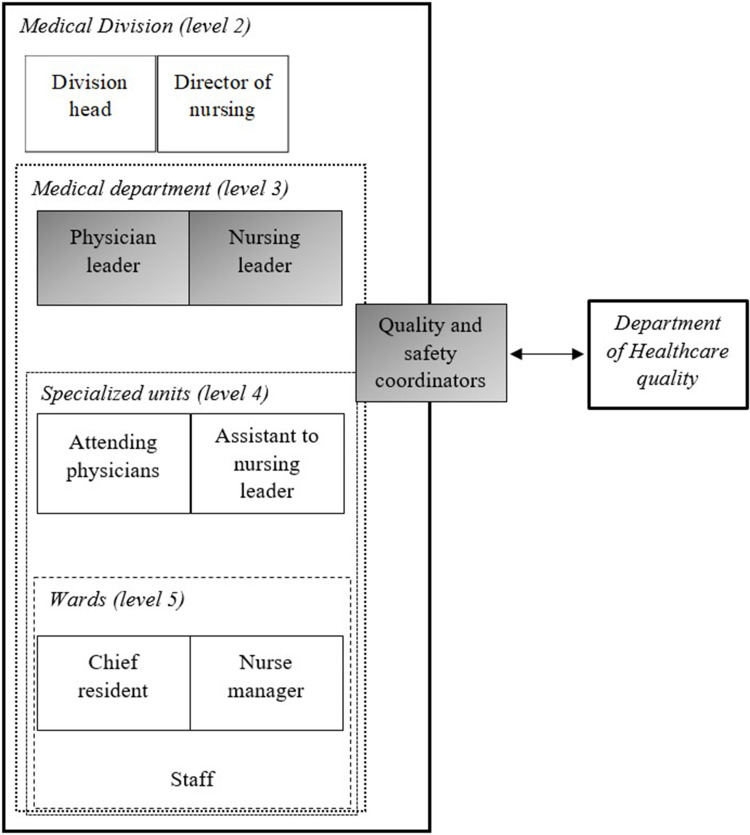
Schematic representation of hierarchical organization of the participants’ position.

**Notes**: For our study, we categorized institutional leaders at levels 1 to 3, encompassing quality and safety coordinators. We referred to levels 4 and 5 for middle managers.

We conducted purposive sampling. The purposive sampling allowed us to ensure representation among the different departments to capture the diverse disciplines and perspectives or strategies in TeamSTEPPS implementation.^25^ KB, who led the TeamSTEPPS implementation project at the time, identified potential participants both implicated in and distant from the TeamSTEPPS implementation. She also identified the different stages and strategies of implementation in the different services. To mitigate bias of her double position, we separated participants identification and contact. KB provided a nominative list of participants which RL used to contact participants and conduct interviews. All participants signed an information and consent form, agreeing to the publication of anonymized responses/direct quotes. Participants were institutional leaders such as physician leaders, nursing leaders and quality and safety coordinators involved in the deployment of TeamSTEPPS in their departments (Figure 1). All participants attended the TeamSTEPPS Mastertrainer training with their own teams.

### Data Collection

We developed a semi-structured interview guide based on a previous study exploring champions’ experiences regarding TeamSTEPPS implementation.^26^ We pre-tested the interview guide with one representative of each group of participants (ie quality coordinators, physician and nursing leaders) to ensure the understanding of questions and choice of wording. We iteratively adapted the interview guide after each pre-test interview, incorporating the participant’s feedback and adapting the guide to her final version (Appendix A). RL recruited the participants via Email invitations with information and consent form (Appendix B). Interviews were audio-recorded and transcribed verbatim. Transcripts were de-identified and recordings were saved on a secure server. The research group regularly discussed the transcripts to ensure homogeneous data collection and analysis. Each author read the transcripts and shared emerging themes with the whole group.

### Data Analysis

We followed the Standards for Reporting Qualitative Research to describe our results.^27^ We used ATLAS.ti 9.1.7.0 for qualitative data coding. We developed our coding in sequential cycles, inspired by the five-phase qualitative analysis process.^28^,^29^ In the first phase, discussion within the research team led to the development of a deductive and inductive codebook (Appendix C). We used TeamSTEPPS conceptual framework as a starting point for the development of the codebook.^23^ First, we used TeamSTEPPS dimensions (ie communication, leadership, mutual support, and situation monitoring) to gather themes deductively. The whole team discussed the coding, ensuring mutual understanding. We iteratively added codes with emerging inductive themes. KB helped to frame the results within their context with her knowledge of the project’s implementation. In a second phase, MCA and RL coded all the transcripts together and iteratively discussed discrepancies. They embedded codes into themes using thematic analysis for results presentation.^30^ During data analysis, illustrative quotes from the transcripts were selected for each theme and gathered into a thematic mind-mapping. Finally, all researchers met together to discuss results’ articulation to ensure mutual understanding and data organization.

We structured our results according to Doise’s levels of analysis.^31^ This analysis indicates that the individual belongs to different spheres of social influence. These influences shape the individual’s identity, behavior and representations. The aim is to understand how these different levels interact mutually (or exclusively) in a given situation for a given individual.^32^ We categorized our themes into the different levels of analysis. We used the intra-individual (the individual himself: skills, attitudes), inter-individual (a group of individuals: actions, interactions), and inter-group (several groups of a common category: roles, functions, status) levels. Inter-categorical level was not assessed since we focused our study on internal implementation processes.

## Results

Of the 15 invited participants, 11 agreed to participate in the study. Reasons for non-participation were lack of time (n=2), non-commitment to the implementation of TeamSTEPPS (n=1) and unmentioned reasons (n=1). Seven participants were female (64%). At the time of the study, four participants were quality and safety coordinators (all females), four were physician leaders (1 female), and three were nursing leaders (2 females).

We structured the presentation of the results following Doise’s levels of analysis. In the first part, we highlighted common themes regarding TeamSTEPPS implementation between the participants. A summary of these results is found in Table 1. In the second part, we highlighted the themes specific to each participants’ positions.

**Table 1:** Common Themes Between Participants Regarding TeamSTEPPS Implementation Categorized by Doise’s Levels of Analysis

Individual Level	Inter-Individual Level	Inter-Group Level
Pedagogical competences Human-centered approach Strategies: Promoting individual responsibility Middle management as change agents Individual support for the collaborator ● Facilitators: Role modeling Active commitment Proactivity and presence on the field ● Barriers: Lack of time Projects overload Projects heterogeneity and lack of shared objectives	Experience with TeamSTEPPS Team building Participation and collaboration Strategies: Healthcare professions’ representation Contextual or local tools’ adaptation Building a project’s core group with key-players ● Facilitators: Champions’ profile and characteristics Implementation sense-making ● Barriers: Institutional silos Ambiguity between multiple roles	Collaborative culture between healthcare professions Professional values Strategies: Individual-institutional synergies Step-by-step implementation Departmental and interdepartmental vision ● Facilitators: Consistency and convergence between institutional projects Institutional communication about the project ● Barriers: Blurred institutional expectations Change in governance

**Note**: Common themes are identified when all professional groups share the same coded segment.

### Individual Level

#### Professional Background and Role Perceptions

At the individual level, participants indicated that their professional background was a key factor in shaping their perspectives about their own role in project management. Physician leaders, often with academic and teaching-oriented backgrounds, tended to approach project management from a more conceptual perspective. They indicated the best practices one could display but seldom referred to the practices they themselves enacted.

Beyond just saying the words, we work in an interprofessional manner, we really bring it to life. And I take that very seriously, as my mission as head of department, because I think that leadership is very influential. And then in terms of interprofessional collaboration and communication, there is a duty to set an example, which I also ask from my managers. Physician leader 4.3

By contrast, nursing leaders and quality coordinators drew on continuous professional development pathways and frontline experience. They demonstrated a more practical understanding of change management for operational and organizational challenges. This difference was reflected in their awareness of the practical implications of project implementation. With their peers, quality coordinators were efficient to highlight potential issues across departments and address them quickly.

So my role is very transversal in that sense; one of my roles, somewhat behind the scenes or in terms of soft skills, is to serve as a link with areas outside the department, such as the intensive care unit, for example. Quality coordinator 6.3

#### Barriers and Facilitators to Commitment

Participants also highlighted several obstacles that influenced their ability to act upon their role perspectives. A recurring and universal theme reported across all professional groups was the lack of dedicated time, as daily clinical and administrative demands often took priority over implementation work. However, the department’s initial decision to engage in TeamSTEPPS created momentum and freed up some time for change efforts. Even if they were simultaneous projects to implement, participants sought after efficiency and resources pooling. This allowed participants to engage meaningfully in the overall change process.

We also changed our department heads, so it’s only been a few months. It’s a huge change. And the visibility I have as a coordinator is that I have insight into all the key institutional programs, like Declic, TeamSTEPPS, Dynamo — and, actually, all of them are connected. […] We’re in a dynamic process of change. I think it will be favorable for the introduction of TeamSTEPPS. Quality coordinator 11.5

Several participants emphasized personal values that underpinned their professional background such openness to change, a willingness to participate actively in innovation processes, and a commitment to human-centered approaches. These values were framed in terms of respect, fairness, and the desire to recognize and empower individuals within the system. To act upon these values, institutional leaders reported the need for personal and professional commitment, describing themselves as proactive agents capable of anticipating needs and driving change.

It’s everyone’s responsibility to ask themselves: ‘Today, is what I’m doing truly aligned with who I am inside?’ It’s about working on that alignment between who I am internally and what I show on the outside. (…). But each of us needs to work on this, and it’s our responsibility to ask ourselves: Does what I’m doing today make sense? Do I enjoy what I do? Do I still take as much pleasure in it? Am I still performing at my best? Nursing leader 1.36

Yet some expressed that their commitment was affected by a perceived lack of identification with the project among staff or participants. Unclearing the absence of clear expectations, employees tended to maintain familiar work habits and routines. This led to a prioritization of individual dynamics, at the expense of standardization and the development of homogenized values. Despite this, participants reported that some TeamSTEPPS tools were easily integrated into daily routines.

#### Areas of Responsibilities

Participants’ responsibilities were shaped by both internal and external expectations. Internal expectations were mostly linked to professional responsibilities or personal standards. External expectations were associated with both the need to promote work routines and the individual’s role as contributor in interprofessional healthcare teams. Nonetheless, participants said that these expectations were frequently hard to meet due to time constraints, competing commitments or managerial instability.

The fragmentation of roles and the overload of projects affected participants’ capacity to meet such expectations. Particularly, some participants indicated a lack of clarity regarding their role and insufficient familiarity with the implementation processes. Also, participants agreed on the critical role of middle management as key driver of implementation. Its proximity to the field positioned it not only as facilitator of change but also as role model, as most of those managers were also TeamSTEPPS champions.

I also think that setting an example is really important. So, in any case, I wouldn’t have wanted to base the approach solely on non-managerial staff — not just because of sustainability concerns, but also because we truly wanted to show that this is an important project within the department. Physician leader 10.14

Expectations on middle management were high in terms of project management and implementation. Some participants described making themselves available to guide and support middle management and teams through change processes.

Performance really depends on the field — we can’t drive performance without it. It’s the people in the field who are the key players, the strong links in the chain. We […] are like crutches, here to support them in moving the system forward. So, if we also have the support of our institution, that’s when we can truly make progress. But we need levers from the field — it’s the people in the field who will be our driving force. Quality coordinator 11.15

Yet they also acknowledged that the continuous demands of daily clinical work, combined with insufficient time or energy, often impeded their ability to sustain motivation and engagement.

### Inter-Individual Level

#### Training and Mutual Support to Foster Alignment

At the inter-individual level, the development of skills and collaboration through the champion training (TeamSTEPPS Mastertrainer training) was perceived as highly beneficial. Participants reported that this training not only enhanced their own competencies but also fostered supportive peer relationships within the service.

One advantage of having trained as a group and as a team […] is that we were all trained in the same tools […] and shared the same mindset. […] In my opinion, that’s what enabled better interprofessional integration. […] So, implementing it collectively gave everyone on the team the same inputs, which then made it easier to roll it out more precisely to the rest of the team. Nursing leader 9.12

These interactions served as a foundation for creating and sustaining project groups built around shared objectives. The emphasis on mutual support and co-learning within these settings contributed to a stronger collective capacity for innovation. Many participants linked the champion training with a broader commitment to teamwork and participative leadership. This facilitated the emergence of an interprofessional alignment in a common objective and the development of partnerships that were essential to the success of the projects. However, participants also reported obstacles in developing a cohesive interprofessional dynamic. Differences in professional cultures, role expectations, and communication style sometimes hindered synergy between team members and complicated efforts toward shared understanding and coordination.

The problem is that TeamSTEPPS training, even if it is a 2-day course, won’t change the way some people communicate. It may make them question their approach, but it won’t change the way they communicate, which is much more deeply rooted in their upbringing, their experiences, and their fears. Quality coordinator 6.26

#### Key Players to Ensure Interprofessional Collaboration

However, participants managed to align visions and foster truly integrated collaboration between professions. They enhanced staff familiarity with TeamSTEPPS by proactively highlighting the alignment between implemented tools, and current working practices and needs.

There were managers as well as people from the field, which worked really well. It meant we could regularly update things using concrete examples. […] And I think one of the real added values was starting from real-life situations from the field, personal experiences and difficulties people faced to show them that, in the end, this could be a solution to existing problems. Physician leader 4.13

Participants highlighted the strategies to ensure professional representativeness within the champions’ teams. A composition reflecting the diversity of professions and departments involved allowed the project to be contextualized to local specificities. The value of this proximity to the field was repeatedly highlighted. It enabled the team to identify and respond to concrete needs while articulating the project in established workplace routines.

What I did was include the field staff in the reflection process […]. And I think that’s what’s really useful — involving people from the ground level right from the start, getting them to think together about the ideal solution, even if I guide them toward an existing tool. Quality coordinator 8.17

The development of a shared sense of purpose was perceived as essential for promoting ownership and ensuring long-term sustainability. Such purpose was enabled by regularly meeting implementation stakeholders from across professions and hierarchical level. Nonetheless, the operationalization of these ideals was not without challenges. Several participants reported that champions were not fully utilized within their respective organizations. It led to role ambiguity and the diffusion of responsibility across collaborators.

Actually, I see this as a challenge we face with other roles as well — the champion doesn’t have a clearly defined mandate, whether within their unit, their department, or at the division-level. […]. They’ve completed the training and have the skills and capacity to support the team through change using the TeamSTEPPS tools, but we are not necessarily going to mobilize their skills and knowledge as champions. Quality coordinator 3.18

Some participants described the importance of a core team of managers with clearly defined roles. This team included both a quality and safety coordinator with a broad strategic overview and middle managers responsible for supporting and overseeing implementation on the ground. When functioning effectively, such a structure facilitated project monitoring and evaluation. However, its success remained contingent on clear communication, shared vision, and mutual recognition of roles across professional boundaries.

### Inter-Group Level

#### Institutional Dynamics and Values

Regarding the inter-group level, the results pointed to a broader institutional dynamic. This dynamic shaped how implementation was understood and enacted across professional and departmental boundaries. Shared professional values such as quality of care and patient safety were often cited as unifying principles across roles and positions. However, the diversity of professional and departmental cultures often posed challenges to alignment and mutual understanding. Several participants highlighted a dual tension: while the institution promoted interprofessional collaboration in principle, its organizational structure remained highly siloed in practice. This dissonance between institutional discourse and operational reality created confusion about roles and expectations. At times, it eroded participants’ confidence in figuring out their position within broader change initiatives.

Today there are medical and economic forces at play, and I’m not sure we’re all moving in the same direction. […] In a department, to promote interprofessional collaboration, we are able to completely centralize or decentralize forces. […] What’s important now is that we remain attentive to ensuring these priorities all serve the same purpose — namely, the patient. Nursing leader 9.24

#### Communication, Coordination and Governance

In this context, participants emphasized the importance of clear, consistent, and practical communication about implementation goals and long-term vision. To counter the loss of momentum, participants highlighted the importance of visible progress and indicators to sustain institutional and staff commitment. When commitment was multidisciplinary, it allowed the development of trust between professions. To this end, the mutual support between healthcare professionals was regularly mentioned as critical dimension.

Ultimately, the ideal would be to trust each other enough to delegate tasks. […] In the spirit of TeamSTEPPS, there’s also a mindset of mutual support and pooling efforts to foster collaboration. Nursing leader 2.27

Some participants noted that institutional communication often prioritized the content of implementation over the implementation processes. It resulted in insufficient preparation for change. This gap was seen as a major barrier to project commitment.

But in a division like diagnostics, which is unique compared to the others and doesn’t function like a care unit, we find ourselves with tools and then have to figure out how to implement them, how… That’s why I think it will take more time. Quality coordinator 6.22

#### Implementation Strategies and Timing

Reported implementation strategies were diverse. It included piloting project (the implementation scales at departmental level), project slicing (the project is implemented in a step-by-step manner), and the cascading or streaming implementation (frontline managers were trained first and expected to disseminate knowledge). Those strategies were generally well-received, but their success was viewed as heavily reliant on the presence of structured guidance, follow-up, and managerial endorsement. One participant described this approach as a “soft revolution”. It reflected a shared preference for gradual change that allows time for adaptation and inclusive processes that foster ownership. Several participants also mentioned the critical role of implementation’s temporality. If the timing was perceived as inadequate due to competing priorities or contextual instability, efforts risked being undermined. Nonetheless, some participants indicated that TeamSTEPPS implementation aligned well with broader structural changes.

When there are changes in governance, it’s difficult to implement projects because you need stable management to be able to follow through. […] I really prefer to let the teams have stable management. Quality coordinator 8.14

Overall, participants sensed the potential benefit of the TeamSTEPPS implementation. They called for a better balance between institutional structure and flexibility. They highlighted that successful implementation depends not only on professional goodwill but also on the organization’s capacity to support, coordinate, and sustain change efforts over time.

#### Professionally Anchored Visions

Participants also mentioned themes which were specific to their position. Some of these themes were shared with another position, but not between all participants’ positions. These themes are summarized for each professional group in Table 2. We highlighted role-specific and shared themes accordingly.

**Table 2:** Shared and Proper Themes Between Participants’ Positions Regarding TeamSTEPPS Implementation Categorized by Doise’s Levels of Analysis

Participants	Type	Individual Level	Inter-Individual Level	Inter-Group Level
Medical leaders N=4, 1 female Services: Rehabilitation Internal medicine Emergency Pharmacy	Proper	Lack of energy or commitment for the project (b)	Developing skills together (f) Generational barriers (b)	Hierarchical verticality (v) Trickle-down effect (s) Lack of a designated project coordinator (b)
	Shared with nursing leader	Openness to change (v) Lack of identification with the project (b)	Celebrate small wins (f)	Interprofessional trust (v)
	Shared with quality coordinators	Being overload with managerial duties (b)	Lack of legitimacy of champions (b) Celebrate small wins (f) Champions’ underutilization (b)	Indicators and reviews (s) Prioritization and standards (s) Lack of change awareness (b) Developing a community of practice (f) Time dedicated to the project (f)
Nursing leaders N=3, 2 females Services: Surgery Psychiatry Private care	Proper	Personal and active implication in the project (f)	Lack of synergies between professions (b) Building competences as a group (f) Field’s receptivity to the implementation (f)	Reinvesting received trainings (c) Paradox between institutional values and departmental silos (b)
	Shared with quality coordinators	Continuing training (c) Personal and professional commitment (v)	Service or department culture (b)	Commitment to healthcare values (v) Using pilot-project (s) Feedback culture (f)
Quality coordinators N=4, 4 females Services: Psychiatry Diagnostic Operations Intensive care	Proper	Being an institutional linkage between departments (f)	Sharing different professional visions (b) Communication not perceived as a competence (b)	Soft revolution (s) Balance between autonomy and institutional support (s) Wreaking institutional silos (f) Inadequate timing for the implementation (b)

**Notes**: Coded clusters: Proper themes are exclusive to one professional group while shared themes are present in at least two groups.

**Abbreviations**: b, barriers; c, compentecies; f, facilitators; s, strategies; v, values.

Medical leaders complained about their marked exposure to institutional and relational obstacles. They referred a lot to their workload when talking about what limited their involvement. Their leverage lied in strategic structuring (indicators, project coordination) and in the search for inter-professional alignment. Nursing leaders demonstrated strong personal and professional commitment that was nurtured by ongoing training and adherence to the institution’s values. Their proximity to the field helped implement change, although they did encounter obstacles linked to a fragmented departmental culture. Quality coordinators considered they played a transversal role in linking professions and hierarchical levels. Although they were the bearers of a strategic vision and a strong commitment, they reported having to cope with a perceived lack of legitimacy, timeframe inadequacies and rigid inter-group boundaries.

## Discussion

We conducted interviews with quality and safety coordinators, physician leaders, and nursing leaders at the Geneva University Hospitals. Using the TeamSTEPPS program as a case study, our findings illustrate the multi-layered conditions necessary for successful innovation in healthcare settings. From individual motivations and competencies to collective strategies and institutional frameworks, the implementation emerges as a negotiated process that relies on coordination, adaptability, and sustained engagement across all levels of the organization.

Participants’ professional background, prior professional experiences and training shaped how they understood and enacted their implementation roles. Consistent with previous studies, their role perception was closely tied to prior professional experiences and training.^7^,^33^ Each professional asserted his/her influence through his/her unique epistemologies and forms of legitimacy.^34^,^35^ In our case, physician leaders approached implementation conceptually, drawing from teaching and academic norms. Nursing leaders and quality coordinators (with nursing backgrounds) brought a practice-oriented view. Practically, this view stemmed from routine problem-solving and hands-on experience. These differences influence both participants’ perception of implementation challenges and the strategies they mobilized in response. These different approaches are complementary and strengthen interprofessional work. At the same time, professional self-conceptions are framed by institutional norms and expectations. This underlines the importance of identifying profession-specific epistemologies of change and distinct sources of legitimacy (strategic authority, relational proximity, transversal coordination). For the individual, these concepts may be aligned or distant. A greater distance creates tensions and limits one’s ability to commit accordingly to personal or professional values. In the context of this study, we highlighted that values such as openness to change and human-centered commitment to care were central, whatever participants’ professional background or position. In our case, such values acted as internal motivational anchors. Aligning these values at the institutional level is critical to manage organizational change.^1^

Yet values alone could not fully compensate for unclear roles or weak institutional support. Despite competing priorities and limited time dedicated to project management, participants emphasized the importance of both personal and collective accountability, and the proactive adaptation of the implementation to their local contexts and needs. They aligned expectations across individual, team and institutional levels. However, implementation overload with fragmentation of roles, unclear expectations or overlapping projects could attenuate their efforts.^36^ This is where middle management emerges as a central cohesive element in the change process.^18^ We highlighted that, when institutional leaders are tied with strategic decisions and governance, middle management may bridge institutional vision and its execution. Participants recognized middle management functions of role-modelling and “pulse-feeling”. This dual role enables middle management to facilitate alignment across organizational levels.^37^ Though, our findings suggest that middle management dual role is possible when protected time, coordination mechanisms and communication are in place. Enabling these elements are the prerogatives of institutional leaders.

Interpersonal collaboration was reinforced by the Mastertrainer training, which was consistently described as empowering and trust-building. Interprofessional and multilevel training has been recognized as a space where silos can be broken down, shared learning can occur, and collective commitment can emerge.^38^,^39^ In our study, training not only enhanced competencies but also created relational bonds and mutual recognition. It provided a platform for identifying local needs and co-developing contextualized solutions, reframing change as a collective endeavor rather than an individual burden. Still, the added value of interprofessional teamwork is not automatically perceived. Participants noted some frictions stemmed from professional cultures or assumptions about roles. These results suggest that developing a shared mental model and a functional communication is a prerequisite for effective collaboration.^40^,^41^ Participants identified that the representation of each profession or position of their department in the team was a key success factor. This allowed for implementation contextualization to specific departmental realities. Teams that reflected diverse professional voices enhanced ownership and relevance of the innovation to implement.^42^ This further commitment and sustainability among staff, leading to a more participative approach that confers authority and clarity.^43^,^44^ In some settings, organizations mitigated tensions by creating core managerial teams combining strategic oversight with operational guidance. Where communication and mutual recognition were strong, these structures enabled cross-boundary cooperation and project monitoring. Conversely, where clarity was lacking, role diffusion and loss of direction ensued.

Beyond the TeamSTEPPS program itself, our findings point to a discrepancy between institutional discourse and organizational reality. While participants valued institutional promotion of interprofessional values and training opportunities, they also described entrenched silos and limited structural follow-through. Such gaps between rhetoric and practice can create confusion, demotivate staff, and fragment accountability.^15^ Participants appreciated the long-term vision and strategic messaging from the institution but emphasized that success depended on embedding implementation within managed, stable, and well-communicated planning processes. In this perspective, structured timelines and visible indicators are recognized as central to maintain momentum and trust.^45^,^46^ Importantly, our results highlighted that both the content and the process of implementation must be prioritized to reduce risks of misalignment or disengagement. In this respect, participants highlighted the critical role of an internal designated coordinator for the implementation. Such individuals who mastered both the content of the innovation and process of the implementation acted as effective boundary-spanners, enhancing organizational readiness and staff confidence. This supports the importance of contextual and relational readiness for change, where individuals dedicated to manage change act as critical success factors.^47^ Participants endorsed gradual implementation strategies such as piloting, project slicing, and cascading, which allowed adaptation over time and across contexts. The “soft revolution” metaphor adequately captured their preference for inclusive, paced change rather than top-down transformation. However, even gradual strategies required structural coordination and managerial commitment to succeed. Finally, participants called for a better balance between institutional structure and flexibility. While professional values and motivation can initiate change, sustained transformation requires organizational scaffolding that allows those values to take root. Implementation success, then, hinges not only on the content of interventions like TeamSTEPPS, but also on the institution’s capacity to adapt its own practices and expectations.

### Practical and Policy Implications

Our findings indicate that sustainable implementation of TeamSTEPPS requires structured alignment across organizational levels. First, aligning institutional ambition and frontline capacity is essential. Participants described tensions between strategic objectives and clinical workload constraints, suggesting that implementation must be synchronized with operational realities. This includes fostering a shared language around collaboration, synchronizing project timelines with workload cycles, and improving horizontal and vertical coordination mechanisms.

Second, reinforcing role clarity and interprofessional literacy is critical. Participants reported difficulties in understanding their own role and others’ expectations. Continuing education initiatives should thus incorporate interprofessional literacy and leadership development to foster shared understanding of roles, responsibilities, and team dynamics. This includes ongoing training and supervision for institutional leaders.

Finally, durable and legitimate support structures are necessary to sustain engagement. It demands durable institutional support structures, including formal mandate definition, visible commitment from institutional leaders, stable coordination teams, role legitimacy, and processes for feedback and follow-up. As the results show, participants often struggled with fragmented guidance and ambiguity in champions positioning.

## Limitations

The study involved 11 participants from a single hospital. Qualitative methodology prioritizes depth and meaning over statistical generalizability.^48^ Thus, the small sample may not fully represent the diversity of perspectives among institutional leaders involved in implementation. Qualitative research often involves self-selection bias, where those more invested or reflective are more likely to participate.^49^ This may result in an overrepresentation of proactive leadership discourses and underrepresentation of more critical or resistant views. However, by using a purposive sampling method, we ensured representativeness for departments, experience with TeamSTEPPS implementation, personal implication, and professions. Secondly, one of the authors (KB) served as co-leader of the TeamSTEPPS project at the HUG. Given her in-depth knowledge of the implementation project, there was a potential risk of bias in theme extraction. To mitigate this, coding was performed independently by RL and MCA. Subsequently discussing the codes and building the themes with the entire research group, we ensured that presented data did not suffer from prior knowledge bias.^50^ Finally, this study was conducted in a single university hospital. It provides a distinct and specific organizational ecosystem: high specialization, academic affiliation, and a structured hierarchy. While this setting offers valuable insight into institutional leadership in tertiary care, the findings may not transfer easily to smaller hospitals, primary care settings, or healthcare systems operating under different governance models. Monocentric designs reflect local organizational cultures and leadership styles.^51^

### Strengths

This study offers several methodological and conceptual strengths that contribute to the field of implementation research and healthcare leadership studies. First, this study is grounded in the exploration of lived experience. By exploring how institutional leaders conceptualize and experience their role in project implementation, this study captures a frame of the complexity of leadership consciousness in healthcare organizations. The qualitative approach allowed participants to reflect openly, yielding rich and sincere data grounded in professional practices.^21^,^22^ Secondly, this study applies a multi-level analytical framework (individual, inter-individual, and inter-group levels) to investigate the layered nature of project implementation.^31^ The framework we chose is rarely used in healthcare leadership research, offering a novel approach to analyze both agency and structure in implementation processes. By integrating theories of role expectation,^34^,^35^ organizational readiness,^47^ and interprofessional collaboration,^39^ the study offers a comprehensive perspective on how leaders operate at the interface between institutional vision and clinical practice. This contributes to ongoing scholarly efforts to re-theorize leadership as a distributed, context-dependent, and relational process rather than an individual or hierarchical attribute.^1^,^52^

### Future Research

The sociocultural context of the French-speaking region of Switzerland plays a significant role in how institutional leadership is conceptualized and enacted. The Swiss healthcare system combines federal decentralization with strong professional autonomy, which can both enable and constrain change processes.^53^ Furthermore, French-speaking institutional cultures tend to emphasize hierarchical authority and formal role boundaries.^54^,^55^ These cultural dynamics may influence, but not only, how institutional managers perceive role legitimacy when leading change; the degree of comfort with interprofessional negotiation or cross-boundary collaboration; and their inclination to engage in distributed leadership versus task-specific coordination.

These factors underscore the importance of contextual reflexivity in implementation research.^56^ Leadership is not only a function of individual capacity but also a product of culturally embedded role expectations, organizational discourses, and institutional norms. Thus, future research may consider exploring the cultural differences influencing implementation leadership. Future work should also investigate how leadership commitment, access to high-quality evidence, and structured decision-making processes interact with cultural contexts to influence leadership effectiveness during implementation.^57^ These may generate valuable recommendations for both institutions and researchers looking for the best suited implementation management for their context.

## Conclusion

This study highlights the interconnected nature of individual, interpersonal, and institutional factors that come into play among institutional leaders when they implement healthcare projects. Successful institutional change depends on the alignment of professional values, interprofessional collaboration, and supportive organizational structures. While shared values such as trust and commitment support interprofessional work, persistent structural and relational barriers such as misalignment between institutional vision and operational reality remain significant challenges. To overcome these obstacles, institutions should not only provide dedicated implementation time and program-specific training but also invest in the development of leaders’ implementation skills (role clarity, coordination capacity) and homogenize governance processes (interprofessional facilitation). Given the inherent complexity of implementation processes, building capacity in areas such as change management, project coordination, and interprofessional facilitation is essential. Isolated interventions suffer from misalignment between individual role perceptions, relational dynamics, and institutional structures. Future initiatives should address these levels simultaneously to achieve context-sensitive and sustainable change.

## Data Availability

Anonymized transcripts (French) are available on request from the corresponding author.
